# Dr. J. N. Brown, Buffalo

**Published:** 1874-09

**Authors:** 


### Obituary.

### Dr. Jeremiah N. Brown.

The members of the Medical Profession of Buffalo, have been called upon to part with another of their fellow members in the person of Dr. J. N. Brown, who died at Utica, September 18th. Dr. Brown had been for some time past suffering from the premonitory symptoms of brain disease, which ended in his death at the Utica Insane Asylum. He graduated at the Buffalo Medical College in 1855, and has since that time been engaged in the practice of his profession in this city. For several years he served as one of the Physicians to the Buffalo General Hospital, and by his devoted attention to his duties and his kind and gentlemanly manner, gained the love and respect of all with whom he came in contact. He devoted himself faithfully to the practice of his profession, and was a noble example of the earnest conscientious physician. He was called away in the full tide of manhood, and his early death will be mourned by a large circle of loving friends.

The following resolutions were adopted at a special meeting of the Erie County Medical Society, held Sept. 19th.

The sad intelligence having come to us of the death, at Utica, of Dr. J. N. Brown, for many years a fellow-member of this society, and convened as we are, to pay fitting respect and honor to the memory, be it, as the sense of this society.

*Resolved*, That by this mournful, yet righteous, providence we are deprived of one of our worthiest members. Studious, unobtrusive, faithful and competent in his professional relations; gentle, kindly and agreeable socially; honorable in all his walks of life, we thus suffer no common loss:

Second, That we proffer our sincere condolence and sympathy to his stricken wife and family. In further token of which we will, as a society, attend his funeral;

Third, That a copy of our action herein be transcribed and sent to the widow, and also be furnished for publication in the daily press and the Buffalo *Medical Journal*.

P. H. STRONG, M. D.,
C. C. F. GAY, M. D.,
JOHN HAUENSTEIN, M. D.

